# Management of pain in patients with bone metastases

**DOI:** 10.3389/fonc.2023.1156618

**Published:** 2023-03-16

**Authors:** Doudou Jing, Qian Zhao, Yibo Zhao, Xiangdong Lu, Yi Feng, Bin Zhao, Xiaofeng Zhao

**Affiliations:** ^1^ Department of Orthopaedics, The Second Hospital of Shanxi Medical University, Taiyuan, China; ^2^ Department of Endocrine, The Second Hospital of Shanxi Medical University, Taiyuan, China

**Keywords:** cancer-induced bone pain, pain management, surgery, conservative therapy, traditional Chinese medicine

## Abstract

Cancer-induced bone pain (CIBP) has a considerable impact on patients’ quality of life as well as physical and mental health. At present, patients with CIBP are managed according to the three-step analgesic therapy algorithm proposed by the World Health Organization. Opioids are commonly used as the first-line treatment for moderate-to-severe cancer pain but are limited due to addiction, nausea, vomiting and other gastrointestinal side effects. Moreover, opioids have a limited analgesic effect in some patients. In order to optimize the management of CIBP, we must first identify the underlying mechanisms. In some patients, surgery, or surgery combined with radiotherapy or radiofrequency ablation is the first step in the management of CIBP. Various clinical studies have shown that anti-nerve growth factor (NGF) antibodies, bisphosphonates, or RANKL inhibitors can reduce the incidence and improve the management of cancer pain. Herein, we review the mechanisms of cancer pain and potential therapeutic strategies to provide insights for optimizing the management of CIBP.

## Introduction

The skeleton is one of the most common metastatic sites in patients with solid tumors such as breast cancer, prostate cancer, kidney cancer and lung cancer (1, 2). The incidence of bone metastasis is associated with considerable economic burden and profound impacts on both physical and mental health. Moreover, patients with bone metastases have significantly lower survival compared with patients without bone metastases (3). With the continuous progress being made in radiotherapy, chemotherapy and surgical treatment, survival time in patients with bone metastases has increased in recent years. However, cancer-induced bone pain (CIBP) is associated with reduced quality of life and negative effects on mental health. Management of CIBP is considered one of most important issues in the treatment of patients with bone metastases. Currently, the recommended strategy is the three-step analgesic therapy pathway proposed by the World Health Organization. Importantly, the effectiveness of this strategy is limited by multiple adverse effects including addiction, nausea, vomiting and other gastrointestinal reactions (4). In addition, some patients experience sub-optimal efficacy. Therefore, how to optimize the management of CIBP has become a hot topic in cancer research.

## Mechanism of bone metastasis

Bone plays a number of complex roles in the body including but not limited to exercise, support and protection of vital organs. Various cells, such as osteoblasts, osteoclasts, adipocytes, and macrophages maintain homeostasis and ensure the basic function of bone (1). Under physiological conditions, the rate of bone resorption and bone formation is finely balanced through the complex interactions between various hormones and cytokines secreted by different types of cell. Menopause and senescence can break balance of bone homeostasis, leading to a series of skeletal related events (SREs). The number of patients experiencing SREs has increased in recent years due to prolonged survival in patients with cancer, in particular breast cancer, lung cancer, prostate cancer, kidney cancer and thyroid cancer (5). Once bone metastasis occurs, tumor cells change the balance of bone absorption and bone reconstruction leading to the formation of metastatic tumors (6). The most likely sites for bone metastasis are regions with a highly active bone marrow microenvironment that promotes cell growth, such as vertebrae (87%), ribs (77%), pelvis (63%), and proximal humerus and femur (3). Bone metastases can be divided into osteolytic lesions, osteogenic lesions, or mixed lesions. However, regardless of the type of lesion, osteoclasts and osteoblasts are both active participants (7).

**Figure 1:**
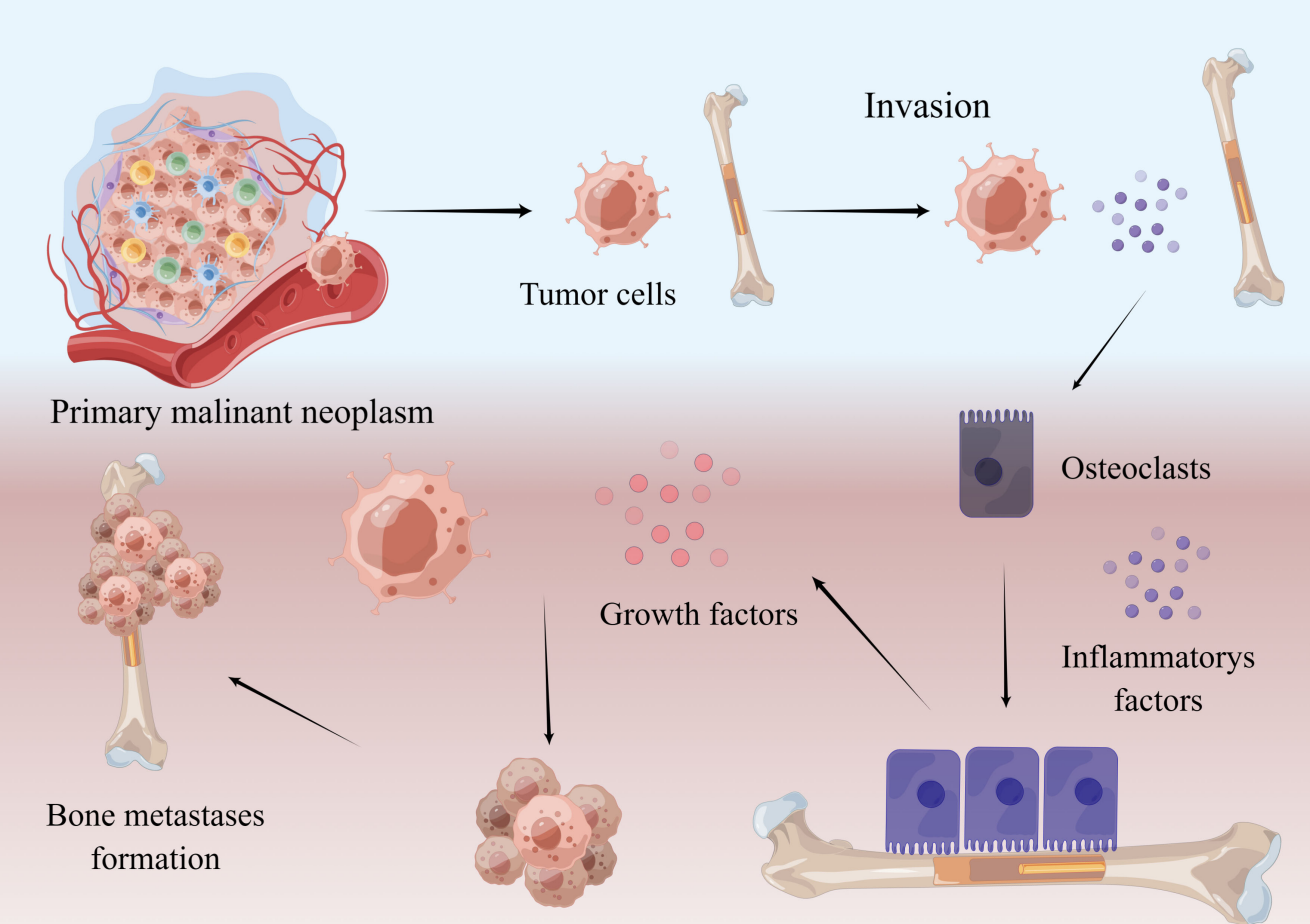
Mechanism of bone metastasis. Malignant neoplasm cells break away from the primary focus and enter the bone. By interacting with other cells, tumor cells secrete RANKL and some inflammatory factors, which promote the maturation of osteoclasts. Osteoclasts absorb bone and release growth factors in bone, which promote tumor growth and bone metastases formation. This figure is designed by Figdraw.

Stephen Paget proposed the hypothesis of “seed and soil” to explain how bone remodeling induces tumor re-implantation and development during bone metastasis. Metastasis from a primary tumor site is a multi-step and multifactorial process, including primary site erosion, breakthrough of basement membrane, escape of nesting apoptosis, and adhesion, colonization and invasion in distant organs (showed in **Figure 1**). Once in the bone, tumor cells promote the secretion of various cytokines such as RANKL, Vascular endothelial growth factor (VEGF), Interleukin-1 (IL-1), Interleukin-6 (IL-6), Interleukin-8 (IL-8), Interleukin-11 (IL-11) and Tumor necrosis factor-α (TNF-α) directly or indirectly, which induces the formation and maturation of osteoclasts resulting in massive bone absorption, which provides favorable conditions for tumor cell colonization (8). In fact, different tumors have different ways to change the balance of local bone metabolism. For example, breast cancer cells secrete parathyroid hormone related peptide (PTHrP) to activate osteoblasts to secrete RANKL and inhibit the expression of osteoprotegerin(OPG), which ultimately leads to increased osteoclast activity and the occurrence of bone metastasis (9). It is also worth noting that activation of bone absorption leads to the release of a large number of growth factors. As a result, these growth factors stimulate the growth of tumor cells, leading to positive feedback to promote tumor growth (10, 11).

## Mechanism of CIBP

CIBP occurs at all stages of bone metastasis and becomes more difficult to control as the degree of tumor growth increases. Research shows that 64% of patients with bone metastasis suffered CIBP, and of those, 75–90% experience severe CIBP (12). CIBP includes background pain, spontaneous pain and occasional (induced) pain. Background pain, aggravated by disease progression, is a continuous dull pain that can usually be controlled by traditional analgesic strategies. Breakthrough cancer pain is extreme pain that is associated with an incidence of 40–81% (13). Breakthrough pain is intermittent, often occurs quickly, and lasts for a short time. Opioids do not adequately control spontaneous pain, and subsequent overuse may be associated with side effects such as nausea, vomiting and respiratory depression (14).

**Figure 2:**
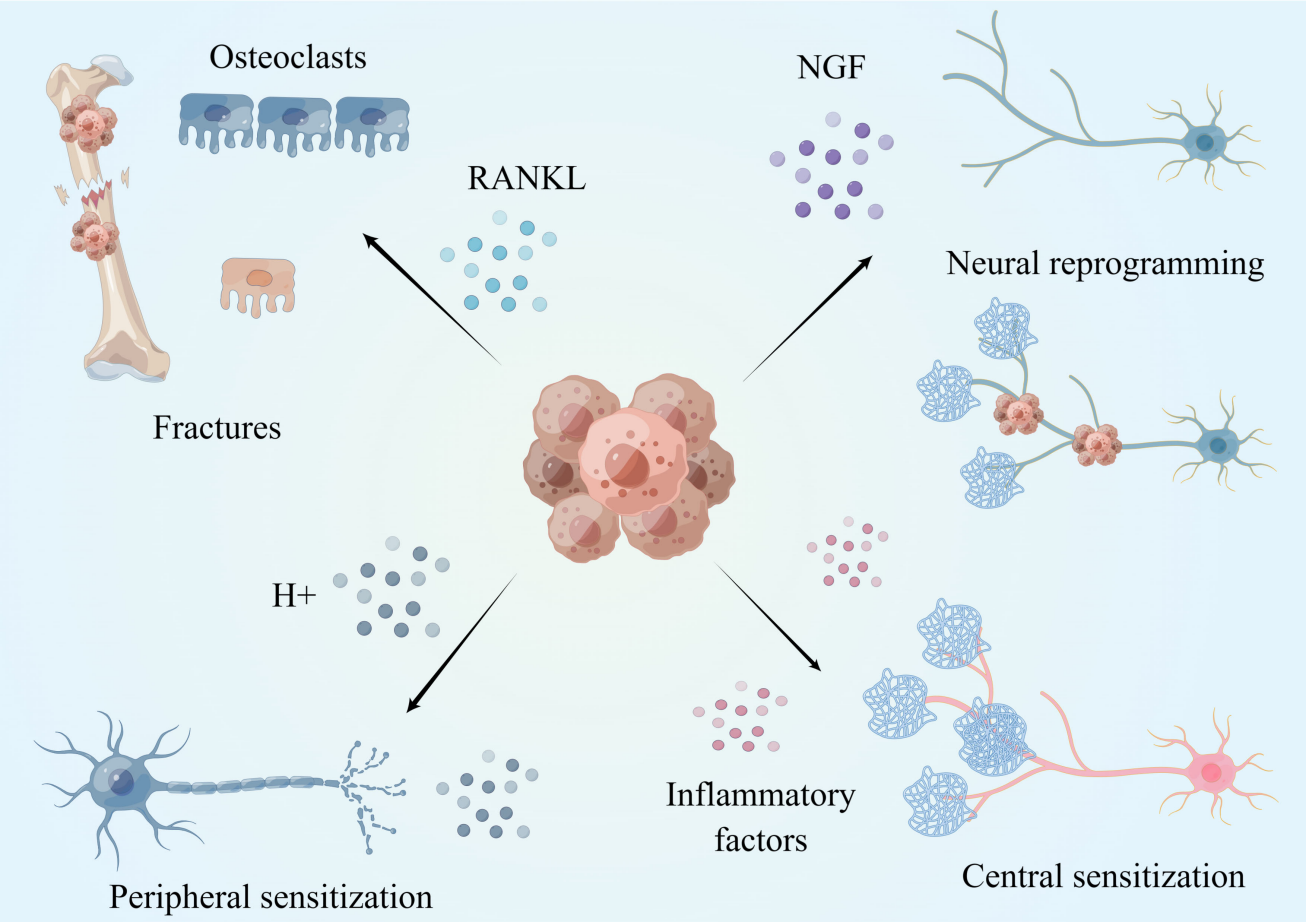
Mechanism of CIBP. Tumor can promote osteoclast maturation and bone resorption by secreting a large amount of RANKL, leading to micro-fracture and pain occurrence. The acidic microenvironment formed by tumor cells can induce peripheral nerve sensitization and promote the occurrence of cancer pain. Tumors induce nerve reprogramming and cancer pain by secreting NGF. Long-term peripheral stimulation and inflammatory factors cause changes in neurons, central sensitization and pain. This figure is designed by Figdraw.

The underlying mechanisms of CIBP may involve inflammatory, ischemic, compressive or injurious neuropathological processes (15) (showed in **Figure 2**). We know that there is a rich distribution of sensory nerves in bone, and most of the nerve fibers are distributed in the periosteum, A-delta and peptic C fibers (TrkA+). There are fewer nerve fibers distributed in the bone marrow and the bone cortex. Once tumor cells coloize bone, they recruit and activate osteoclasts and osteoblasts. Combined with highly active bone resorption and bone formation, bone metastases grow and proliferate. During this process, the imbalance of bone homeostasis induced by tumor cells leads to the occurrence of micro fractures. Micro fractures activate nociceptors, which leads to pain. The malignant growth of tumor tissue is associated with compression, which can also stimulate nociceptive sensory neurons (16). Additionally, with malignant growth of bone metastasis, various immune cells are recruited into the tumor including macrophages, T cells, and NK cells. Massive inflammatory factors or mediators, including prostaglandin E2 (PGE2), Nerve growth factor (NGF), bradykinin and proinflammatory cytokines (TNF-α, IL-1β, IL-6, IL-8, IL-15), chemokines (CCL5), monocyte chemoattractant protein-1 (MCP-1), macrophage inflammatory protein-1a (MIP-1a), and extracellular adenosine triphosphate (ATP), are secreted by various immune cells following interaction with tumor cells. Research shows that inflammatory factors can directly activate receptors located in sensory nerve fibers, including the endothelin receptor (ETAR), prostaglandin (PG) receptor, TrkA receptor, bradykinin receptor, cytokine receptor, chemokine receptor, transient receptor potential channel, vanillin subfamily member 1(TRPV1), acid sensing ion channel 3 (ASIC3) and purinergic receptor (P2X3), resulting in CIBP (17).

Tumor cells also induce nerve sprouting and promote the growth of sensory nerves and sympathetic nerves into tumor tissue, resulting in CIBP. NGF is an essential neurotrophic factor that induces the growth of sensory and sympathetic nerves, which is highly expressed in tumor cells and various immune cells in the tumor microenvironment (TME). NGF is commonly identified in prostate cancer, gastric cancer, and breast cancer (18, 19). Recently, a study showed that high expression of NGF is closely related to CIBP (20). Exogenous NGF application, overexpression of NGF, and inhibition of NGF degradation can induce mechanical pain and thermal hyperalgesia in animal models of pain. Inhibition of NGF and its receptors can significantly inhibit the development of tumors and related spontaneous and induced pain behaviors (21–25). It also has been shown that NGF can activate nociceptors and ion channels (such as P2X3, TRPV1, ASIC-3) by up-regulating the expression of a variety of proteins such as substance P, calcitonin gene related peptide (CGRP), and bradykinin, resulting in CIBP (17). More importantly, NGF can induce neural remodeling. In normal tissues, the sensory and sympathetic nerves are separated. However, tumor cells induce and drive the generation and growth of axons and nerves by secreting high levels of NGF. As a result, by increasing the density of nerve fibers, NGF also stimulates sensory nerve fibers and sympathetic nerve fibers to produce connections and form a neuroma-like structure in the tumor, known as Neural reprogramming (26). It is clear that the formation of neuroma-like structures in tumor tissues is closely related to CIBP (27–29). In the prostate bone metastatic tumor model, multiple nerve fibers were observed, mixing with prostate cancer cells and related stromal cells, to form a beaded neuroma-like structure (30). Anti-NGF treatment significantly reduced the density of nerve fibers, the formation of neuroma-like structures, the frequency of CIBP, and the generation of tumor-induced nociceptive behavior. It has been reported that anti-NFG treatment can also reduce bone damage caused by sarcoma (31, 32). In addition to NGF, tumors also secrete other neurotrophic substances such as brain derived neurotrophic factor (BDNF), which is closely related to cancer pain (33–35).

In addition to directly activating peripheral nociceptors, the low pH and hyperinflammatory state of the TME will lead to the remodeling of nociceptors in tumor tissue. Long-term stimulation with H+ ions and sustained inflammatory factors continuously activates nociceptors, leading to increased sensitivity of nociceptive neurons and amplification of their afferent nerve signals, which called as peripheral sensitization. Once peripheral sensitization occurs, as a result, even if the stimulation is not enough to trigger nociceptive signal transduction, this can still result in pain signal transduction in patients with cancer (36). Continuous activation of peripheral nociceptors can also lead to central sensitization.

The complex microenvironment in tumor tissue is associated with long-term and high-intensity activation of peripheral high-density nociceptors and induce changes in the central nervous system through a variety of mechanisms, which is manifested in the increased responsiveness to peripheral stimuli, thus generating central sensitization(Central sensitization refers to the abnormal increase of excitability or enhancement of synaptic transmission of pain-related neurons in the spinal cord and above, including the increase of spontaneous discharge activity of neurons, the expansion of sensory domain, the reduction of threshold to external stimuli, the enhancement of response to suprathreshold stimuli and other pathological changes, thus amplifying the transmission of pain signals. Its corresponding clinical manifestations include spontaneous pain, hyperalgesia and allodynia.) and inducing cancer pain. Studies have shown that under stimulation by tumor tissue, glial cells in the spinal cord are activated, especially spinal microglia and astrocytes. The overactive state of spinal microglia and astrocytes is one of the key factors that activates central sensitization and leads to CIBP (2, 37–40). Glial cells also promote CIBP by secreting interleukin 1 (IL-1) and interleukin 18 (IL-18) (41, 42). Multiple targets and signal pathways in spinal cord glial cells, such as protein deacetylase (HDACs) (39), epidermal growth factor receptor (EGFR) and MCP-1 (43), have been shown to be involved in the formation of CIBP through central sensitization. Glial cells located near the periaqueductal gray matter (PAG) of the midbrain are also activated in tumor-bearing mice, and a several cytokines such as IL-1 and IL-6 are secreted to activate the PI3K-AKT pathway in PAG, leading to central sensitization and cancer pain. Blocking this signal pathway can reduce mechanical and thermal hyperalgesia in rats with bone cancer (44).

It is estimated that 20–85% of patients who receive neurotoxic chemotherapy will experience peripheral neuropathy (15, 45–47). The usage of platinum-based antineoplastic agents, vinca alkaloids, epothilones (ixabepilone), taxanes, proteasome inhibitors (bortezomib) and immunomodulatory drugs (thalidomide) often results in the development of peripheral neuropathy, which may be accompanied by changes in motor and autonomic nerve function (48). The mechanism of peripheral neuropathy may involve mitochondrial dysfunction and oxidative stress, the release of inflammatory mediators (cytokines and chemical factors), ion channel dysfunction and intracellular signal transduction. However, the mechanisms remain to be fully elucidated (49).

Overall, the mechanisms underlying the occurrence and deterioration of CIBP are complex and involve tumor implantation, bone metabolism, enhancement of mechanical stimulation, activation and alteration of peripheral nerves, peripheral nerve remodeling, and activation/adaptive changes in the central nervous system. It will be vital to understand these mechanisms to facilitate the optimal management of CIBP in practice.

## Pain management of bone metastases

Management of CIBP in patients with bone metastasis is an important issue, and the strategy of analgesia accompanies the life of patients, which is involved the three-ladder model of analgesia proposed by the World Health Organization

Nonopioids, such as nonsteroidal anti-inflammatory drugs (NSAIDs) are often the first-line therapy for CIBP. They are effective against mild-to-moderate pain; however, there is little evidence for the effectiveness of NSAIDs in patients with CIBP (50). While NSAIDs may inhibit cancer-related pain in some patients, they are unlikely to provide gain additional benefits in patients with moderate-to-severe CIBP who have already been treated with strong opioids. Furthermore, the long-term use of NSAIDs has been associated with cardiovascular and gastrointestinal risks (51). Some studies have shown that NSAIDs may have limited anti-tumor effects; however, there is a lack of robust clinical data to support these conclusions (52).

Weak opioids, such as tramadol and codeine, are not commonly used to manage CIBP because there is limited evidence for their effectiveness. Low-dose morphine is associated with more rapid and effective analgesic effects than opioids (53, 54).

For severe CIBP, strong oral and percutaneous opioids are the first choice for patients. Studies have shown that buprenorphine, oxycodone, fentanyl and morphine have similar analgesic effects and gradually achieve complete analgesia with adequate drug supply. Morphine is the first-line treatment for breakthrough pain and is associated with effective pain control (55). However, research shows that prolonged use of strong opioids is associated with side effects such as nausea, lethargy, vomiting and constipation (56). Detrimental effects of opioids on the liver and kidney compound the side effect of opioids. Epidural intrathecal injection of opioids can significantly relieve cancer pain; however, this type of pain management requires administration by healthcare professional and may be expensive.

Methadone is an effective substitute for morphine, oxycodone, fentanyl and other opioid drugs, which shows incomplete cross tolerance with other opioid receptors. However, due to significant inter-individual differences in the plasma half-life of methadone, it should be used only under the supervision of professional doctors. Although morphine and methadone show similar analgesic effects after single dose administration, it is recommended to reduce the anodynia dose by one quarter to one twelfth when switching from another opioid to methadone to prevent side effects such as respiratory depression (57).

### Surgery

In patients with a long-life expectancy and no important organ metastasis, surgical resection is the first choice for patients with bone metastases with only one site metastasis.

For other metastatic spine tumors, patients with unstable spine, spinal cord compression or nerve function injury, tumor resection and reconstruction surgery are also should be chose firstly, for reducing compression on the spinal cord, which can relieve local pain by improving neural function (58). Various scoring systems such as the revised Tokuhashi scoring system and Tomita scoring system indicate estimated prognosis and appropriate treatment strategies in different patients (59). The evaluation indicators of these systems include patients’ general condition, the number of bone metastases outside the spine, the number of vertebral bodies involved, and whether the metastases in key organs are resectable. Based on these comprehensive scoring systems, surgeons can predict each patient’s survival period and determine the optimal treatment method. In fact, timely surgical intervention can fully relieve CIBP. Encouragingly, scoring systems are constantly improving. There is also the possibility that algorithms could be developed for specific tumor types to further improve the accuracy of scoring. VEGF, EGFR and other molecular markers in tumor tissue can also be included in scoring systems to provide more accurate estimations of survival time and therapeutic effect (60, 61). Spinal separation surgery is an option for patients with bone metastases who cannot tolerate total spine resection. After surgery, radiotherapy should be administered for maximum anti-tumor effects (62). For patients with bone metastasis without spinal cord compression and spinal instability, minimally invasive spinal surgery (Percutaneous vertebroplasty, PVP or Percutaneous kyphoplasty, PKP) may be considered. The heat released by bone cement during surgery can kill local nerves and tumor tissue and effectively relieve the pain associated with thoracolumbar metastatic tumors. Post-operative adjuvant radiotherapy can also be effective in these patients (63, 64).

For long-bone metastasis, the Mirels predictive score is widely used in clinical practice (65). Based on this score, the optimal treatment strategy is identified according to metastasis location, the severity of pain, X-ray findings, and the invasion rate of lesions. In keeping with the tumor-free principle, biological reconstruction is the main goal of this surgery and pain can be significantly relieved using this method (66). Three-dimensional printing technology may be a new choice for reconstruction after resection. It can be used to print complex structures that are difficult to fabricate using traditional processes and overcome the problems of stress shielding and low biological activity of conventional prostheses (67).

For pelvis metastasis, there is no widely recognized scoring system. Because the pelvic anatomy is complex and adjacent to important organs and blood vessels, the resection and reconstruction of pelvic metastasis must be performed by experienced surgeons. While this surgery can help to relieve CIBP, the effects of such a complex surgery can be associated with additional pain.

### Radiotherapy

Radiotherapy is a safe and effective strategy to relieve CIBP who are not suitable or cannot tolerate surgical treatment. Radiotherapy can significantly relieve CIBP. Studies have shown that 60% of patients with bone metastases experience significant pain relief after radiotherapy. Intraoperative radiotherapy is associated with particularly effective control of pain; however, it is worth noting that radiotherapy may lead to the occurrence of fracture or nerve injury in the spinal cord (68). Compared with ordinary radiotherapy, stereotactic body radiotherapy (SBRT) can deliver high-dose radiation to tumors while protecting adjacent normal tissues. Research shows that the local control rate of SBRT can reach 90%, and the probability of vertebral fracture and nerve injury after radiotherapy can be significantly reduced (69, 70). Evidence shows that more than 80% of patients can achieve significant remission of CIBP in a few days. Importantly, the safety of spinal SBRT depends on the tolerance of adjacent organs, especially the spinal cord. On the premise of ensuring the safety of the spinal cord, the radiation dose can be appropriately increased to avoid recurrence at the outer edge of the tumor target area. In clinical application, the strategy that radiotherapy combined with other therapies is common in manage CIBP in bone metastasis. Compared with radiotherapy alone, those strategy has a higher pain relief rate (71, 72).

### Radiofrequency ablation

Radiofrequency ablation (RFA) includes microwave ablation and low-temperature ablation. RFA can be combined with radiotherapy and surgery. Research shows that RFA is associated with effective local pain control in around 64–77% of cases, which can relieve 70–100% of the pain related to bone metastases. After RFA treatment, PVP or PKP can prevent fracture and stabilize the surgical effect (73). However, the application of RFA must be carefully considered particularly in cases where structures such as the spinal cord, main nerves and blood vessels are within 1 cm of the tumor.

### Bone-targeted therapy

Bisphosphonates are pyrophosphate analogues that combine with hydroxyapatite on the bone surface, directly inhibiting the attachment, differentiation and maturation of osteoclasts, which reduces the rate of bone absorption. Bisphosphonates have become the standard of care for bone metastases and have been shown to reduce the incidence of hypercalcemia and the rate of SREs. Studies have shown that regardless of whether radiotherapy and chemotherapy are combined, bisphosphonate treatment reduces the occurrence of CIBP (74). However, it is worth noting that bisphosphonates do not have a significant inhibitory effect on acute pain.

The RANKL/RANK signal pathway is a central to the regulation of osteoclast differentiation and activation. RANKL activates RANK binding on the surface of osteoclast precursor cells, and promotes osteoclast maturation. Denosumab, the most widely used RANKL inhibitor, prevents RANKL from combining with RANK, thus inhibiting the formation and activation of osteoclasts, result in reduced bone absorption and increased bone mass. Treatment with denosumab has been shown to inhibit tumor metastasis (75–77).

It is worth mentioning that many studies have found that compared with bisphosphonates, Denosumab have more advantages in reducing SREs and alleviating pain (76, 78, 79).

### NGF inhibitor

Many humanized anti-NGF antibodies have shown encouraging results in clinical trials. Moreover, anti-NGF antibodies has a significant inhibitory effect on CIBP, especially neural cancer pain. Additionally, experiments in mice show that NGF antibody treatment reduces bone damage caused by tumors, delays fracture time, and prolongs the use of tumor-carrying limbs, although the underlying mechanisms of these effects are unknown (32). Both exercise and weight bearing can promote bone health. However, there are no studies on the ability of NGF to promote the recovery of patients’ motor function. Once bone metastases are diagnosed, some patients decline surgery since the chance of removing all tumor cells is very slim. As a result, NSAIDs and weak opioids are commonly used as first-line treatment according to the principles of three steps of cancer medication. After cancer pain develops to a certain extent, strong opioids are often used to control the pain. Since the growth of nerves and the formation of peripheral sensitization may be implicated in the early stages of bone metastasis formation, there is limited potential for successful treatment with anti-NGF therapy. At present, we could not identify any studies evaluating the effectiveness of anti-NGF therapy for the prevention of CIBP.

### Anticonvulsants and antidepressants

Neuropathic pain is an important component of CIBP. Therefore, in recent years, pregabalin has been increasingly used in the treatment of CIBP and is particularly effective especially for medium- and short-term CIBP (80, 81). The efficacy of gabapentin in the treatment of CIBP is limited.

Antidepressants including amitriptyline and duloxetine can also be used for the treatment of neuropathic pain. Data show that both of these drugs can help to improve quality of life in patients with CIBP (12).

### Traditional Chinese medicine

Traditional Chinese medicine can be used to treat pain, including CIBP. The advantages of traditional Chinese medicine include rich dosage forms, light toxicity and side effects, and improved tolerability compared with more established methods. For these reasons, it has gained traction as a treatment option for patients with CIBP. Studies have shown that astragalus, psoralen, scutellaria barbata, atractylodes macrocephala, and corydalis can significantly inhibit CIBP in patients with cancer (82–85). Insect drugs, such as scorpion and Huchansu have also been shown to be effective for the management of CIBP (86–88).

Acupuncture is effective for alleviating pain and can help to reduce the need for opioids (89). Electrical stimulation with acupuncture combined with opioids has potential to reduce the side effects associated with opioids and improve quality of life in patients with CIBP (90–92). Research shows that acupuncture activates sympathetic nerve fibers to increase endogenous opioids at inflammatory sites to inhibit pain. Various inflammatory factors such as β- Endorphin and 5-HT are implicated in the relief of pain through acupuncture, both at the peripheral and central level (93). Injection of acupoints can also reduce the frequency of cancer pain (94).

Although the mechanisms of treating cancer pain with traditional Chinese medicine are not yet fully elucidated, it is clear that this form of medicine has unique advantages for treating cancer pain compared with more commonly used methods. Going forward, it will be important to understand the mechanisms by which Chinese medicine is able to reduce CIBP, and to standardize usage and dosage to allow more widespread use.

### Other therapy

Injection of a TNF-α antagonist can partly block the mechanical hyperalgesia in oral cancer (95, 96). In addition, recent studies show that the application of some immune agents, such as anti-PD-L1 monoclonal antibodies, Sting inhibitors, and *in vitro* immune cell adoptive therapy can also relieve the pain caused by primary tumors and bone metastases (97–100).

## Conclusions

The management of CIBP is a complex issue for patients and physicians. Active pain management, such as early surgical intervention for eligible patients. Radiotherapy and microwave therapy can be combined with surgical intervention to obtain a higher pain relief rate and promote the patient to recover the patient’s nerve and activity function as soon as possible. Early intervention with RANKL inhibitors, bisphosphonates or anti-NGF antibodies in patients who are not candidates for surgery may improve quality of life. Traditional Chinese medicine and acupuncture also have the potential to become important options for CIBP management. Overall, there is no single solution for managing CIBP in all patients. Only by fully elucidating the underlying mechanisms of cancer pain will it be possible to optimize the management of CIBP in real-world practice.

## Author contributions

DJ and ZQ conceived the manuscript and drafted the manuscript. YBZ and XDL contributed to data collection. YF, BZ, and XFZ reviewed the manuscript. All authors contributed to the article and approved the submitted version.

## References

[1] Fornetti J Welm AL Stewart SA. Understanding the bone in cancer metastasis. J Bone Miner Res (2018) 33:2099–113. doi: 10.1002/jbmr.3618 30476357

[2] Chen SP Sun J Zhou YQ Cao F Braun C Luo F. Sinomenine attenuates cancer-induced bone pain *via* suppressing microglial JAK2/STAT3 and neuronal CAMKII/CREB cascades in rat models. Mol Pain (2018) 14:1744806918793232. doi: 10.1177/1744806918793232 30027795 PMC6096675

[3] Coleman RE Croucher PI Padhani AR Clezardin P Chow E Fallon M. Bone metastases. Nat Rev Dis Primers (2020) 6:83. doi: 10.1038/s41572-020-00216-3 33060614

[4] Zajaczkowska R Kocot-Kepska M Leppert W Wordliczek J. Bone pain in cancer patients: Mechanisms and current treatment. Int J Mol Sci (2019) 20(23):6047. doi: 10.3390/ijms20236047 31801267 PMC6928918

[5] Li B Wang P Jiao J Wei H Xu W Zhou P. Roles of the RANKL-RANK axis in immunity-implications for pathogenesis and treatment of bone metastasis. Front Immunol (2022) 13:824117. doi: 10.3389/fimmu.2022.824117 35386705 PMC8977491

[6] Clezardin P Coleman R Puppo M Ottewell P Bonnelye E Paycha F. Bone metastasis: mechanisms, therapies, and biomarkers. Physiol Rev (2021) 101:797–855. doi: 10.1152/physrev.00012.2019 33356915

[7] Yang M Liu C Yu X. Skeletal-related adverse events during bone metastasis of breast cancer: current status. Discov Med (2019) 27:211–20. 31361984

[8] Gdowski AS Ranjan A Vishwanatha JK. Current concepts in bone metastasis, contemporary therapeutic strategies and ongoing clinical trials. J Exp Clin Cancer Res (2017) 36:108. doi: 10.1186/s13046-017-0578-1 28800754 PMC5554406

[9] Othman A Winogradzki M Lee L Tandon M Blank A Pratap J. Bone metastatic breast cancer: Advances in cell signaling and autophagy related mechanisms. Cancers (Basel) (2021) 13(17):4310. doi: 10.3390/cancers13174310 34503118 PMC8431094

[10] Norregaard KS Jurgensen HJ Gardsvoll H Engelholm LH Behrendt N Soe K. Osteosarcoma and metastasis associated bone degradation-a tale of osteoclast and malignant cell cooperativity. Int J Mol Sci (2021) 22(13):6865. doi: 10.3390/ijms22136865 34202300 PMC8269025

[11] Hiraga T. Bone metastasis: Interaction between cancer cells and bone microenvironment. J Oral Biosci (2019) 61:95–8. doi: 10.1016/j.job.2019.02.002 31109867

[12] Scarborough BM Smith CB. Optimal pain management for patients with cancer in the modern era. CA Cancer J Clin (2018) 68:182–96. doi: 10.3322/caac.21453 PMC5980731 29603142

[13] Lohre ET Thronaes M Klepstad P. Breakthrough cancer pain in 2020. Curr Opin Support Palliat Care (2020) 14:94–9. doi: 10.1097/SPC.0000000000000494 32332210

[14] Davies AN. Breakthrough cancer pain. Curr Pain Headache Rep (2014) 18:420. doi: 10.1007/s11916-014-0420-9 24760489

[15] Feller L Khammissa RAG Bouckaert M Ballyram R Jadwat Y Lemmer J. Pain: Persistent postsurgery and bone cancer-related pain. J Int Med Res (2019) 47:528–43. doi: 10.1177/0300060518818296 PMC6381470 30632434

[16] Bortolin A Neto E Lamghari M. Calcium signalling in breast cancer associated bone pain. Int J Mol Sci (2022) 23(3):1902. doi: 10.3390/ijms23031902 35163823 PMC8836937

[17] Hirose M Kuroda Y Murata E. NGF/TrkA signaling as a therapeutic target for pain. Pain Pract (2016) 16:175–82. doi: 10.1111/papr.12342 26452158

[18] Pundavela J Roselli S Faulkner S Attia J Scott RJ Thorne RF. Nerve fibers infiltrate the tumor microenvironment and are associated with nerve growth factor production and lymph node invasion in breast cancer. Mol Oncol (2015) 9:1626–35. doi: 10.1016/j.molonc.2015.05.001 PMC5528785 26009480

[19] Griffin N Faulkner S Jobling P Hondermarck H. Targeting neurotrophin signaling in cancer: The renaissance. Pharmacol Res (2018) 135:12–7. doi: 10.1016/j.phrs.2018.07.019 30031169

[20] Yoneda T Hiasa M Okui T Hata K. Sensory nerves: A driver of the vicious cycle in bone metastasis? J Bone Oncol (2021) 30:100387. doi: 10.1016/j.jbo.2021.100387 34504741 PMC8411232

[21] Slatkin N Zaki N Wang S Louie J Sanga P Kelly KM. Fulranumab as adjunctive therapy for cancer-related pain: A phase 2, randomized, double-blind, placebo-controlled, multicenter study. J Pain (2019) 20:440–52. doi: 10.1016/j.jpain.2018.09.014 30368018

[22] Kumar V Mahal BA. NGF - the TrkA to successful pain treatment. J Pain Res (2012) 5:279–87. doi: 10.2147/JPR.S33408 PMC3442742 23028238

[23] Bimonte S Cascella M Forte CA Esposito G Cuomo A. The role of anti-nerve growth factor monoclonal antibodies in the control of chronic cancer and non-cancer pain. J Pain Res (2021) 14:1959–67. doi: 10.2147/JPR.S302004 PMC8253925 34234542

[24] Han L Jiang J Xue M Qin T Xiao Y Wu E. Sonic hedgehog signaling pathway promotes pancreatic cancer pain *via* nerve growth factor. Reg Anesth Pain Med (2020) 45:137–44. doi: 10.1136/rapm-2019-100991 31792027

[25] Guedon JG Longo G Majuta LA Thomspon ML Fealk MN Mantyh PW. Dissociation between the relief of skeletal pain behaviors and skin hypersensitivity in a model of bone cancer pain. Pain (2016) 157:1239–47. doi: 10.1097/j.pain.0000000000000514 PMC5142607 27186713

[26] Buehlmann D Ielacqua GD Xandry J Rudin M. Prospective administration of anti-nerve growth factor treatment effectively suppresses functional connectivity alterations after cancer-induced bone pain in mice. Pain (2019) 160:151–9. doi: 10.1097/j.pain.0000000000001388 30161041

[27] Lucchesi M Lanzetta G Antonuzzo A Rozzi A Sardi I Favre C. Developing drugs in cancer-related bone pain. Crit Rev Oncol Hematol (2017) 119:66–74. doi: 10.1016/j.critrevonc.2017.08.005 28893462

[28] Park SH Eber MR Fonseca MM Patel CM Cunnane KA Ding H. Usefulness of the measurement of neurite outgrowth of primary sensory neurons to study cancer-related painful complications. Biochem Pharmacol (2021) 188:114520. doi: 10.1016/j.bcp.2021.114520 33741328 PMC8154668

[29] Silverman DA Martinez VK Dougherty PM Myers JN Calin GA Amit M. Cancer-associated neurogenesis and nerve-cancer cross-talk. Cancer Res (2021) 81:1431–40. doi: 10.1158/0008-5472.CAN-20-2793 PMC7969424 33334813

[30] Jimenez-Andrade JM Ghilardi JR Castaneda-Corral G Kuskowski MA Mantyh PW. Preventive or late administration of anti-NGF therapy attenuates tumor-induced nerve sprouting, neuroma formation, and cancer pain. Pain (2011) 152:2564–74. doi: 10.1016/j.pain.2011.07.020 PMC3199350 21907491

[31] Bloom AP Jimenez-Andrade JM Taylor RN Castaneda-Corral G Kaczmarska MJ Freeman KT. Breast cancer-induced bone remodeling, skeletal pain, and sprouting of sensory nerve fibers. J Pain (2011) 12:698–711. doi: 10.1016/j.jpain.2010.12.016 21497141 PMC3111843

[32] McCaffrey G Thompson ML Majuta L Fealk MN Chartier S Longo G. NGF blockade at early times during bone cancer development attenuates bone destruction and increases limb use. Cancer Res (2014) 74:7014–23. doi: 10.1158/0008-5472.CAN-14-1220 PMC4253026 25287160

[33] Ostrow KL Donaldson KJ Caterina MJ Belzberg A Hoke A. The secretomes of painful versus nonpainful human schwannomatosis tumor cells differentially influence sensory neuron gene expression and sensitivity. Sci Rep (2019) 9:13098. doi: 10.1038/s41598-019-49705-w 31511601 PMC6739480

[34] Grayson M Arris D Wu P Merlo J Ibrahim T Fang-Mei C. Oral squamous cell carcinoma-released brain-derived neurotrophic factor contributes to oral cancer pain by peripheral tropomyosin receptor kinase b activation. Pain (2022) 163:496–507. doi: 10.1097/j.pain.0000000000002382 34321412 PMC8678394

[35] Zhang Y Lin C Wang X Ji T. Calcitonin gene-related peptide: A promising bridge between cancer development and cancer-associated pain in oral squamous cell carcinoma. Oncol Lett (2020) 20:253. doi: 10.3892/ol.2020.12116 32994816 PMC7509602

[36] Duitama M Moreno Y Santander SP Casas Z Sutachan JJ Torres YP. TRP channels as molecular targets to relieve cancer pain. Biomolecules (2021) 12(1):1. doi: 10.3390/biom12010001 35053150 PMC8774023

[37] Chen YL Feng XL Cheung CW Liu JA. Mode of action of astrocytes in pain: From the spinal cord to the brain. Prog Neurobiol (2022) 219:102365. doi: 10.1016/j.pneurobio.2022.102365 36228888

[38] Midavaine E Cote J Marchand S Sarret P. Glial and neuroimmune cell choreography in sexually dimorphic pain signaling. Neurosci Biobehav Rev (2021) 125:168–92. doi: 10.1016/j.neubiorev.2021.01.023 33582232

[39] He XT Hu XF Zhu C Zhou KX Zhao WJ Zhang C. Suppression of histone deacetylases by SAHA relieves bone cancer pain in rats *via* inhibiting activation of glial cells in spinal dorsal horn and dorsal root ganglia. J Neuroinflamm (2020) 17:125. doi: 10.1186/s12974-020-01740-5 PMC7175547 32321538

[40] Meng W Hao MM Yu N Li MY Ding JQ Wang BH. 2-bromopalmitate attenuates bone cancer pain *via* reversing mitochondrial fusion and fission imbalance in spinal astrocytes. Mol Pain (2019) 15:1744806919871813. doi: 10.1177/1744806919871813 31394961 PMC6710711

[41] Huo W Zhang Y Liu Y Lei Y Sun R Zhang W. Dehydrocorydaline attenuates bone cancer pain by shifting microglial M1/M2 polarization toward the M2 phenotype. Mol Pain (2018) 14:1744806918781733. doi: 10.1177/1744806918781733 29882480 PMC6009085

[42] Liu S Liu YP Lv Y Yao JL Yue DM Zhang MY. IL-18 contributes to bone cancer pain by regulating glia cells and neuron interaction. J Pain (2018) 19:186–95. doi: 10.1016/j.jpain.2017.10.003 29079540

[43] Zheng XQ Wu YH Huang JF Wu AM. Neurophysiological mechanisms of cancer-induced bone pain. J Adv Res (2022) 35:117–27. doi: 10.1016/j.jare.2021.06.006 PMC8721251 35003797

[44] Li Y Bao Y Zheng H Qin Y Hua B. The nonreceptor protein tyrosine kinase src participates in every step of cancer-induced bone pain. BioMed Pharmacother (2021) 141:111822. doi: 10.1016/j.biopha.2021.111822 34147901

[45] Velasco R Navarro X Gil-Gil M Herrando-Grabulosa M Calls A Bruna J. Neuropathic pain and nerve growth factor in chemotherapy-induced peripheral neuropathy: Prospective clinical-pathological study. J Pain Symptom Manage (2017) 54:815–25. doi: 10.1016/j.jpainsymman.2017.04.021 28797868

[46] Khasabova IA Seybold VS Simone DA. The role of PPARgamma in chemotherapy-evoked pain. Neurosci Lett (2021) 753:135845. doi: 10.1016/j.neulet.2021.135845 33774149 PMC8089062

[47] Sarezky J Sachs G Elinzano H Stavros K. Cancer and peripheral nerve disease. Clin Geriatr Med (2021) 37:289–300. doi: 10.1016/j.cger.2021.01.003 33858611

[48] Zajaczkowska R Kocot-Kepska M Leppert W Wrzosek A Mika J Wordliczek J. Mechanisms of chemotherapy-induced peripheral neuropathy. Int J Mol Sci 20 (2019) 20(6):1451. doi: 10.3390/ijms20061451 PMC6471666 30909387

[49] Shim HS Bae C Wang J Lee KH Hankerd KM Kim HK. Peripheral and central oxidative stress in chemotherapy-induced neuropathic pain. Mol Pain (2019) 15:1744806919840098. doi: 10.1177/1744806919840098 30857460 PMC6458664

[50] Strawson J. Nonsteroidal anti-inflammatory drugs and cancer pain. Curr Opin Support Palliat Care (2018) 12:102–7. doi: 10.1097/SPC.0000000000000332 29432225

[51] Thiruchenthooran V Sanchez-Lopez E Gliszczynska A. Perspectives of the application of non-steroidal anti-inflammatory drugs in cancer therapy: Attempts to overcome their unfavorable side effects. Cancers (Basel) (2023) 15(2):475. doi: 10.3390/cancers15020475 36672424 PMC9856583

[52] Mercadante S Casuccio A Agnello A Pumo S Kargar J Garofalo S. Analgesic effects of nonsteroidal anti-inflammatory drugs in cancer pain due to somatic or visceral mechanisms. J Pain Symptom Manage (1999) 17:351–6. doi: 10.1016/S0885-3924(98)00141-9 10355213

[53] Chapman EJ Edwards Z Boland JW Maddocks M Fettes L Malia C. Practice review: Evidence-based and effective management of pain in patients with advanced cancer. Palliat Med (2020) 34:444–53. doi: 10.1177/0269216319896955 31980005

[54] Wiffen PJ Derry S Moore RA. Tramadol with or without paracetamol (acetaminophen) for cancer pain. Cochrane Database Syst Rev (2017) 5:CD012508. doi: 10.1002/14651858.CD012508 28510996 PMC6481722

[55] Alarcon MDL Estevez FV Cabezon-Gutierrez L Padros MC Martin-Arroyo JMT Rebollo MA. Expert consensus on the management of breakthrough cancer pain in older patients. A Delphi study. J Geriatr Oncol (2019) 10:643–52. doi: 10.1016/j.jgo.2019.03.012 31036463

[56] Nosek K Leppert W Nosek H Wordliczek J Onichimowski D. A comparison of oral controlled-release morphine and oxycodone with transdermal formulations of buprenorphine and fentanyl in the treatment of severe pain in cancer patients. Drug Des Devel Ther (2017) 11:2409–19. doi: 10.2147/DDDT.S141007 PMC5574595 28860712

[57] Fallon M Giusti R Aielli F Hoskin P Rolke R Sharma M. Management of cancer pain in adult patients: ESMO clinical practice guidelines. Ann Oncol (2018) 29:iv166–91. doi: 10.1093/annonc/mdy152 30052758

[58] Stewart CL Warner S Ito K Raoof M Wu GX Kessler J. Cytoreduction for colorectal metastases: liver, lung, peritoneum, lymph nodes, bone, brain. when does it palliate, prolong survival, and potentially cure? Curr Probl Surg (2018) 55:330–79. doi: 10.1067/j.cpsurg.2018.08.004 PMC6422355 30526930

[59] Tokuhashi Y Uei H Oshima M. Classification and scoring systems for metastatic spine tumors: a literature review. Spine Surg Relat Res (2017) 1:44–55. doi: 10.22603/ssrr.1.2016-0021 31440612 PMC6698555

[60] Gregory TM Coriat R Mir O. Prognostic scoring systems for spinal metastases in the era of anti-VEGF therapies. Spine (Phila Pa 1976) (2013) 38:965–6. doi: 10.1097/BRS.0b013e3182609d38 22648032

[61] Yang M Xu W Liu T Yang X Wang P Wu S. Development and validation of a novel survival prediction model in patients with spinal metastasis from non-small cell lung cancer. Spine (Phila Pa 1976) (2019) 44:246–57. doi: 10.1097/BRS.0000000000002816 30059487

[62] Di Perna G Cofano F Mantovani C Badellino S Marengo N Ajello M. Separation surgery for metastatic epidural spinal cord compression: A qualitative review. J Bone Oncol (2020) 25:100320. doi: 10.1016/j.jbo.2020.100320 33088700 PMC7559860

[63] Pezeshki PS Davidson S Murphy K McCann C Slodkowska E Sherar M. Comparison of the effect of two different bone-targeted radiofrequency ablation (RFA) systems alone and in combination with percutaneous vertebroplasty (PVP) on the biomechanical stability of the metastatic spine. Eur Spine J (2016) 25:3990–6. doi: 10.1007/s00586-015-4057-0 26206290

[64] Wu W Zhang X Li X Liu H Xu L Liu T. Comparison of the clinical outcomes of percutaneous kyphoplasty for the management of osteolytic and osteoblastic-related metastatic vertebral lesions. J Neurointerv Surg (2022) 14:938–41. doi: 10.1136/neurintsurg-2021-018007 34475252

[65] Crenn V Carlier C Gouin F Sailhan F Bonnevialle P members of the So.F.C.O.T. High rate of fracture in long-bone metastasis: Proposal for an improved mirels predictive score. Orthop Traumatol Surg Res (2020) 106:1005–11. doi: 10.1016/j.otsr.2020.03.034 32782175

[66] Pu F Zhang Z Wang B Liu J Shao Z. En bloc resection and intercalary prosthesis implantation for the treatment of humeral diaphyseal bone metastases. Int Orthop (2021) 45:281–8. doi: 10.1007/s00264-020-04845-x 33025082

[67] Pu F Wu W Jing D Yu Y Peng Y Liu J. Three-dimensional-printed titanium prostheses with bone trabeculae enable mechanical-biological reconstruction after resection of bone tumours. Biomater Transl (2022) 3:134–41. doi: 10.12336/biomatertransl.2022.02.005 PMC9465991 36105569

[68] Tsukamoto S Kido A Tanaka Y Facchini G Peta G Rossi G. Current overview of treatment for metastatic bone disease. Curr Oncol (2021) 28:3347–72. doi: 10.3390/curroncol28050290 PMC8482272 34590591

[69] De la Pinta C. SBRT in non-spine bone metastases: a literature review. Med Oncol (2020) 37:119. doi: 10.1007/s12032-020-01442-1 33221952

[70] Zeng KL Tseng CL Soliman H Weiss Y Sahgal A Myrehaug S. Stereotactic body radiotherapy (SBRT) for oligometastatic spine metastases: An overview. Front Oncol (2019) 9:337. doi: 10.3389/fonc.2019.00337 31119099 PMC6504814

[71] Yamada K Kaise H Taguchi T Horiguchi J Takao S Suzuki M. Strontium-89 plus zoledronic acid versus zoledronic acid for patients with painful bone metastatic breast cancer. J Bone Miner Metab (2022) 40:998–1006. doi: 10.1007/s00774-022-01366-y 36042056

[72] Lu CW Shao J Wu YG Wang C Wu JH Lv RX. Which combination treatment is better for spinal metastasis: Percutaneous vertebroplasty with radiofrequency ablation, 125I seed, zoledronic acid, or radiotherapy? Am J Ther (2019) 26:e38–44. doi: 10.1097/MJT.0000000000000449 29087367

[73] Yevich S Chen S Metwalli Z Kuban J Lee S Habibollahi P. Radiofrequency ablation of spine metastases: A clinical and technical approach. Semin Musculoskelet Radiol (2021) 25:795–804. doi: 10.1055/s-0041-1740351 34937119

[74] He F Matsumoto Y. Basic and clinical associations between bone and cancer. Immunol Med (2020) 43:103–6. doi: 10.1080/25785826.2020.1754084 32301686

[75] Cleeland CS Body JJ Stopeck A von Moos R Fallowfield L Mathias SD. Pain outcomes in patients with advanced breast cancer and bone metastases: results from a randomized, double-blind study of denosumab and zoledronic acid. Cancer-Am Cancer Soc (2013) 119:832–8. doi: 10.1002/cncr.27789 22951813

[76] Smith MR Coleman RE Klotz L Pittman K Milecki P Ng S. Denosumab for the prevention of skeletal complications in metastatic castration-resistant prostate cancer: comparison of skeletal-related events and symptomatic skeletal events. Ann Oncol (2015) 26:368–74. doi: 10.1093/annonc/mdu519 PMC4304378 25425475

[77] Dorff TB Agarwal N. Bone-targeted therapies to reduce skeletal morbidity in prostate cancer. Asian J Androl (2018) 20:215–20. doi: 10.4103/aja.aja_12_18 PMC5952474 29553053

[78] Chen F Pu F. Safety of denosumab versus zoledronic acid in patients with bone metastases: A meta-analysis of randomized controlled trials. Oncol Res Treat (2016) 39:453–9. doi: 10.1159/000447372 27487236

[79] Zhang Z Pu F Shao Z. The skeletal-related events of denosumab versus zoledronic acid in patients with bone metastases: A meta-analysis of randomized controlled trials. J Bone Oncol (2017) 9:21–4. doi: 10.1016/j.jbo.2017.09.003 PMC5671384 29123990

[80] Gul SK Tepetam H Gul HL. Duloxetine and pregabalin in neuropathic pain of lung cancer patients. Brain Behav (2020) 10:e01527. doi: 10.1002/brb3.1527 31967742 PMC7066365

[81] Fallon MT. Neuropathic pain in cancer. Br J Anaesth (2013) 111:105–11. doi: 10.1093/bja/aet208 23794652

[82] Su P Leng Y Liu J Yu Y Wang Z Dang H. Comparative analysis of the efficacy and safety of different traditional Chinese medicine injections in the treatment of cancer-related pain: A Bayesian network meta-analysis. Front Pharmacol (2021) 12:803676. doi: 10.3389/fphar.2021.803676 35197850 PMC8858830

[83] Luo Y Wang CZ Sawadogo R Tan T Yuan CS. Effects of herbal medicines on pain management. Am J Chin Med (2020) 48:1–16. doi: 10.1142/S0192415X20500019 32054304

[84] Perez AT Arun B Tripathy D Tagliaferri MA Shaw HS Kimmick GG. A phase 1B dose escalation trial of scutellaria barbata (BZL101) for patients with metastatic breast cancer. Breast Cancer Res Treat (2010) 120:111–8. doi: 10.1007/s10549-009-0678-5 20054647

[85] Chen Y Cheng CS Tan HY Tam CW Wang N Feng Y. Efficacy of herbal medicines intervention for colorectal cancer patients with chemotherapy-induced gastrointestinal toxicity - a systematic review and meta-analysis. Front Oncol (2021) 11:629132. doi: 10.3389/fonc.2021.629132 33869014 PMC8044744

[86] Richard SA Kampo S Sackey M Hechavarria ME Buunaaim ADB. The pivotal potentials of scorpion buthus martensii karsch-Analgesic-Antitumor peptide in pain management and cancer. Evid Based Complement Alternat Med (2020) 2020:4234273. doi: 10.1155/2020/4234273 33178316 PMC7647755

[87] Yu J Luo Y Jin H Lv J Zhou T Yabasin IB. Scorpion alleviates bone cancer pain through inhibition of bone destruction and glia activation. Mol Pain (2020) 16:1744806920909993. doi: 10.1177/1744806920909993 32052691 PMC7054730

[88] Xu J Qian SS Chen YG Li DY Yan Q. [Systematic review and meta-analysis of efficacy and safety of huachansu in treating cancer-related pain]. Zhongguo Zhong Yao Za Zhi (2019) 44:2627–36. doi: 10.19540/j.cnki.cjcmm.20190304.003 31359733

[89] He Y Guo X May BH Zhang AL Liu Y Lu C. Clinical evidence for association of acupuncture and acupressure with improved cancer pain: A systematic review and meta-analysis. JAMA Oncol (2020) 6:271–8. doi: 10.1001/jamaoncol.2019.5233 PMC6990758 31855257

[90] Liang Y Bao G Gong L Zhou J Kong X Ran R. Evaluating the analgesic effect and advantage of transcutaneous electrical acupoint stimulation combined with opioid drugs for moderate to severe cancer-related pain: a study protocol for a randomized controlled trial. Trials (2019) 20:40. doi: 10.1186/s13063-018-3145-y 30635007 PMC6329064

[91] Dai L Liu Y Ji G Xu Y. Acupuncture and derived therapies for pain in palliative cancer management: Systematic review and meta-analysis based on single-arm and controlled trials. J Palliat Med (2021) 24:1078–99. doi: 10.1089/jpm.2020.0405 33691493

[92] He L Tan K Lin X Yi H Wang X Zhang J. Multicenter, randomized, double-blind, controlled trial of transcutaneous electrical nerve stimulation for pancreatic cancer related pain. Med (Baltimore) (2021) 100:e23748. doi: 10.1097/MD.0000000000023748 PMC7870216 33592831

[93] Zhang R Lao L Ren K Berman BM. Mechanisms of acupuncture-electroacupuncture on persistent pain. Anesthesiology (2014) 120:482–503. doi: 10.1097/ALN.0000000000000101 24322588 PMC3947586

[94] Cheon S Zhang X Lee IS Cho SH Chae Y Lee H. Pharmacopuncture for cancer care: a systematic review. Evid Based Complement Alternat Med (2014) 2014:804746. doi: 10.1155/2014/804746 24899911 PMC4036607

[95] Scheff NN Ye Y Bhattacharya A MacRae J Hickman DN Sharma AK. Tumor necrosis factor alpha secreted from oral squamous cell carcinoma contributes to cancer pain and associated inflammation. Pain (2017) 158:2396–409. doi: 10.1097/j.pain.0000000000001044 PMC5680143 28885456

[96] Salvo E Tu NH Scheff NN Dubeykovskaya ZA Chavan SA Aouizerat BE. TNFalpha promotes oral cancer growth, pain, and schwann cell activation. Sci Rep (2021) 11:1840. doi: 10.1038/s41598-021-81500-4 33469141 PMC7815837

[97] Wang K Gu Y Liao Y Bang S Donnelly CR Chen O. PD-1 blockade inhibits osteoclast formation and murine bone cancer pain. J Clin Invest (2020) 130:3603–20. doi: 10.1172/JCI133334 PMC7324182 32484460

[98] Wang K Donnelly CR Jiang C Liao Y Luo X Tao X. STING suppresses bone cancer pain *via* immune and neuronal modulation. Nat Commun (2021) 12:4558. doi: 10.1038/s41467-021-24867-2 34315904 PMC8316360

[99] Zhou X Qiao G Ren J Wang X Wang S Zhu S. Adoptive immunotherapy with autologous T-cell infusions reduces opioid requirements in advanced cancer patients. Pain (2020) 161:127–34. doi: 10.1097/j.pain.0000000000001702 31568023

[100] Ji RR Chamessian A Zhang YQ. Pain regulation by non-neuronal cells and inflammation. Science (2016) 354:572–7. doi: 10.1126/science.aaf8924 PMC5488328 27811267

